# *PPARevolution*. First PPARδ Agonist and a Dual PPARα/PPARδ Activator Approved for the Treatment of Primary Biliary Cholangitis

**DOI:** 10.1111/jcmm.70948

**Published:** 2025-11-13

**Authors:** Iuliana Popescu

**Affiliations:** ^1^ Barnstable Brown Diabetes Center University of Kentucky Lexington Kentucky USA

Primary biliary cholangitis (PBC) is a chronic disease of a cholestatic nature (more predominant in middle‐aged women) resulting from the progressive autoimmune destruction and apoptosis of cholangiocytes in the small intrahepatic bile ducts. This results in the over‐accumulation of bile acids (BA), which further damages the bile ducts and hepatocytes [1]. Although PBC is a rare liver condition, if left untreated, it significantly affects the quality of life (jaundice, pruritus, abdominal pain, fatigue) and severely impairs liver function, leading to liver fibrosis, cirrhosis and cancer. *UDCA (ursodeoxycholic acid)* is the first‐line standard treatment of PBC. However, ~40% of patients do not completely respond to the drug. Therefore, *obeticholic acid (OCA)*, a synthetic bile acid that, unlike UDCA, binds to the FXR nuclear receptor, was approved by the FDA in 2016 as a second‐line therapy (in monotherapy or associated with UDCA); although OCA has proven anti‐fibrotic and anti‐inflammatory properties, its efficacy is limited in decompensated cirrhosis and because of side effects (i.e., pruritus) [2].

## 1 Novel PPAR Agonists as Second‐Line Treatment of PBC

Among the new therapeutic opportunities, Peroxisome Proliferator‐Activated Receptor (PPAR) agonists and SPPARM (selective PPAR modulators) molecules are considered one of the most promising options currently being researched.

**FIGURE 1:**
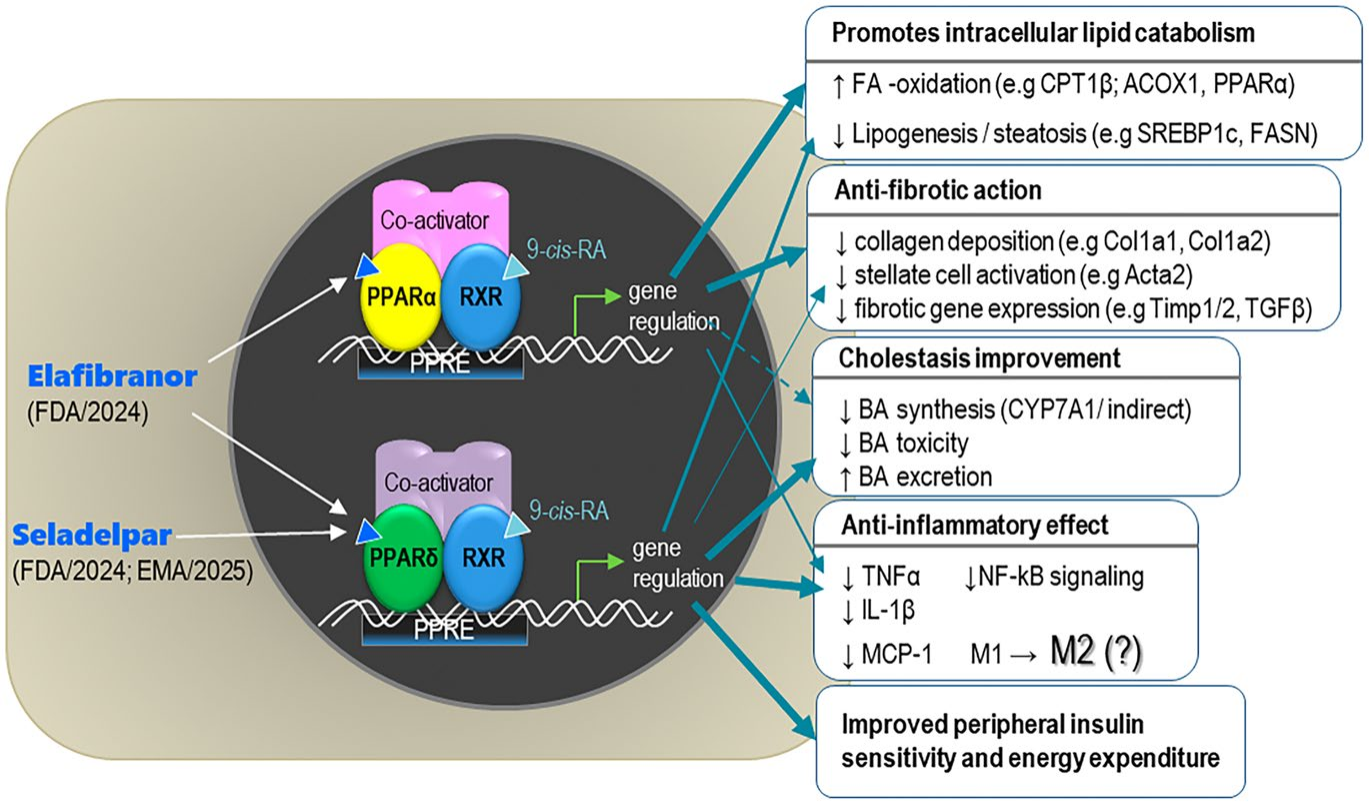
Molecular mechanism of *seladelpar* and *elafibranor* and the main, experimentally demonstrated molecular/cellular actions. Several examples of genes consistently regulated by the two drugs are shown for each therapeutic effect.

The family of PPARs comprises three ligand‐activated nuclear receptors—PPARα, PPARβ/δ and PPARγ (encoded by three different genes)—which have emerged as therapeutic targets in metabolic syndrome, type 2 diabetes, dyslipidaemia and inflammation (fibrates and thiazolidinediones have been developed as classical activators of PPARα and PPARγ, respectively). All three PPAR isotypes behave as endogenous fatty acid sensors and transducers of nutritional stimuli into changes in gene expression. Ligand‐activated PPARs control the transcription of target genes by binding (as heterodimers with the retinoid‐X‐receptor RXR, in super‐complexes with recruited co‐activators) to specific PPAR‐responsive elements (PPRE) located in the promoter region of target genes (Figure 1). Therefore, the therapeutic effects of *seladelpar* (accelerated approval by FDA/2024 and EMA/2025) and *elafibranor* (accelerated approval by FDA/2024) rely on the regulation (up‐ or down‐regulation) of gene sets controlling metabolic, anti‐inflammatory and anti‐fibrotic mechanisms (Figure 1).

## 2 Seladelpar, the First Selective PPARδ Agonist Approved as a Therapeutic Agent

Until recently, PPARβ/δ was not a target of a marketed drug, but several synthetic ligands (e.g., GW501516, GW7042, L165041) have been developed over time and widely used in preclinical research (and some of them also in clinical trials) to understand the mechanisms and therapeutic impact of PPARδ activation in various tissues. PPARδ is ubiquitously expressed, with relatively high levels in metabolically active tissues—muscle, adipose tissue and liver (hepatocytes, Kupffer cells and cholangiocytes), where it exerts many therapeutic effects based on its capacity to transcriptionally control a panel of metabolic genes responsible for lipid and glucose metabolism, BA biosynthesis and the inflammatory phenotype of macrophages [3]. The selective PPARδ agonist *seladelpar* reduces BA accumulation in human and mouse hepatocytes by repressing (via an indirect FGF21‐dependent mechanism) the transcription of the CYP7A1 gene, the rate‐limiting enzyme for BA biosynthesis [4]; this mechanism can explain why the total BA pool size is reduced by *seladelpar* in patients with PBC [5]. In both murine and human hepatocytes, *seladelpar* strongly induces the mRNA expression of PDK4 [4], a direct target gene of PPARδ, suggesting a negative effect on glucose utilisation in hepatocytes (similar to skeletal muscle) and a switch to FA oxidation as an energy fuel [6] (CPT‐1/2 gene controlling mitochondrial FA β‐oxidation is a PPARδ target gene). In a diet‐induced model of MASH, treatment with *seladelpar* alone or in combination with liraglutide (a GLP‐1 analogue) or selonsertib (an investigational ASK1 protein inhibitor), substantially reduced liver fibrosis and steatosis (compared to OCA). The mechanisms underlying these effects involve the activation of gene sets that promote peroxisomal and mitochondrial lipid oxidation [6]. The clearance of lipids from hepatocytes appears to be common among all PPAR agonists and may involve the activation of autophagy [7]. The metabolic role of PPARδ activation in cholangiocytes (a direct effect on FA oxidation or BA transport) is *not* well established. Although not clearly demonstrated for *seladelpar*, it can be speculated that some of its beneficial effects observed in clinical trials may stem from a PPARδ‐induced shift from a pro‐inflammatory M1 to an anti‐inflammatory M2 phenotype of Kupffer cells [8], and possibly, to the suppression of the polarisation of type 1 and 17 helper T cells [9], which may be beneficial in reducing the autoimmunity in PBC. Overall, the clinical evidence gathered to date supports the effectiveness of *seladelpar* in reducing biochemical markers of liver damage, such as alkaline phosphatase (ALP) and total bilirubin, as well as significantly alleviating cholestatic pruritus in patients with PBC [10].

## 3 Elafibranor, a Dual PPARα/PPARδ Agonist With Broad Metabolic Benefits

Elafibranor combines the therapeutic benefits of PPARα activation (robust hepatic lipid clearance) with the peripheral metabolic and anti‐inflammatory benefits of PPARδ agonism, producing broader protection against metabolic and cholestatic injury [11].

PPARα is primarily expressed in the liver, as well as in the kidney, heart and skeletal muscle, where it regulates fatty acid oxidation and glucose homeostasis. It is also expressed in the endothelial cells, vascular smooth muscle cells and macrophages, where it exerts anti‐inflammatory and anti‐oxidant effects. Hepatic PPARα controls many genes of lipid metabolism, in particular those implicated in mitochondrial and peroxisomal β‐oxidation, fatty acid and lipoprotein synthesis and transport, rendering it a molecular target in dyslipidemia, atherosclerosis and MASH. Apart from dietary mono‐ and polyunsaturated fatty acids, PPARα is activated by synthetic ligands such as the fibrates (e.g., fenofibrate, gemfibrozil, bezafibrate), therapeutic molecules prescribed to lower plasma TG and improve HDL‐C. PPARα activation in hepatocytes also modulates the transcription of genes involved in BA metabolism: it represses BA biosynthesis, reduces BA toxicity and promotes BA hepatobiliary excretion (anti‐cholestatic effect) [12].

Transcriptomic results obtained in NASH models of human primary hepatocytes and stem‐cell‐derived progenitors treated in vitro with *elafibranor* evidenced some opposing mechanistic effects regarding its anti‐steatosis effect [13]. As a result, the molecule was withdrawn from late clinical trials for the treatment of adults with NASH and fibrosis, but it demonstrated statistically significant improvements in biomarkers of PBC progression [11]. In animal models, *elafibranor* reduced hepatic lipid accumulation, blunted inflammatory signalling, improved insulin sensitivity, stimulated hepatocellular lipid catabolism/autophagy and ultimately reduced stellate‐cell activation and fibrosis [14, 15, 16]. There is no direct evidence that *elafibranor* suppresses BA synthesis in hepatocytes or modulates the expression of canonical BA transporters in human hepatocytes or cholangiocytes. Clinical and preclinical data show improved BA biochemistry after *elafibranor* administration, an effect that can be indirectly attributed to the δ component shared by *elafibranor* and *seladelpar*. Preclinical data demonstrated that *elafibranor* may reduce the expression of inflammatory cytokines/chemokines (TNFα, IL‐1β, MCP‐1) and dampen the TLR4/NF‐κB pathway in hepatic immune cells [13, 14]. Evidence from mouse model studies also reports the polarisation of macrophage phenotype toward a less pro‐inflammatory M2 state [17, 18] and it might also suppress the differentiation of helper CD4^+^ T cell‐type 17 [19], underlying mechanisms by which it can lower hepatic and systemic inflammation [1] and autoimmunity in MASH and cholestatic models. However, direct evidence of *elafibranor*‐induced M2 polarisation in human Kupffer cells from patients is absent.

In summary, both *seladelpar* and *elafibranor* improve liver biochemistry, reduce cholestasis, inflammation and fibrosis, enhance fatty acid oxidation and metabolic efficiency, and have favourable lipid‐modulating and insulin‐sensitizing effects (Figure 1).

## 4 Quo Tendimus?

Although both molecules improve biochemical markers of PBC (ALP, total bilirubin), some experts argue these markers reflect a surrogate endpoint rather than direct clinical outcomes. *Seladelpar* has demonstrated a consistent beneficial effect on pruritus; however *elafibranor*'s effect on pruritus is modest (it might be related to a smaller affinity of this molecule for PPARδ). In fact, in the absence of long‐term comparative outcome data, it's still difficult to conclude which profile offers the greater real‐world benefit on disease modification. Also, safety data in special conditions or populations (pregnancy, children, older age) need to be further investigated, as well as possible organ toxicity risk and interactions with other drugs at a systemic level.

The next steps in PBC therapy focus on personalised, combination treatments that target both cholestasis and symptoms—new and old pan‐PPAR agonists (pemafibrate, bezafibrate), dual agonists PPARα/PPARγ (e.g., saroglitazar, although other ‘glitazars’ have been withdrawn from studies because of paradoxical cardiac adverse effects), FXR, PXR and FGF19 modulators, inhibitors of the ASBT transporter, antifibrotic agents and modulators of the gut microbiota [20], to improve biochemical response, quality of life and long‐term outcomes beyond the UDCA therapy.

## Author Contributions

**Iuliana Popescu:** conceptualization, writing – original draft, review and editing.

## Conflicts of Interest

The author declares no conflicts of interest.

## Data Availability

The author has nothing to report.
